# Plant Species Rather Than Climate Greatly Alters the Temporal Pattern of Litter Chemical Composition During Long-Term Decomposition

**DOI:** 10.1038/srep15783

**Published:** 2015-10-30

**Authors:** Yongfu Li, Na Chen, Mark E. Harmon, Yuan Li, Xiaoyan Cao, Mark A. Chappell, Jingdong Mao

**Affiliations:** 1 Zhejiang Provincial Key Laboratory of Carbon Cycling in Forest Ecosystems and Carbon Sequestration, Zhejiang A & F University, Lin’an 311300, China; 2 Department of Chemistry and Biochemistry, Old Dominion University, 4541 Hampton Blvd, Norfolk, VA 23529, USA; 3 Department of Forest Ecosystems and Society, Oregon State University, Corvallis, OR 97331, USA; 4 Environmental Laboratory, U.S. Army Corps of Engineers, 3909 Halls Ferry Rd., Vicksburg, MS 39180, USA

## Abstract

A feedback between decomposition and litter chemical composition occurs with decomposition altering composition that in turn influences the decomposition rate. Elucidating the temporal pattern of chemical composition is vital to understand this feedback, but the effects of plant species and climate on chemical changes remain poorly understood, especially over multiple years. In a 10-year decomposition experiment with litter of four species (*Acer saccharum*, *Drypetes glauca*, *Pinus resinosa*, and *Thuja plicata*) from four sites that range from the arctic to tropics, we determined the abundance of 11 litter chemical constituents that were grouped into waxes, carbohydrates, lignin/tannins, and proteins/peptides using advanced ^13^C solid-state NMR techniques. Decomposition generally led to an enrichment of waxes and a depletion of carbohydrates, whereas the changes of other chemical constituents were inconsistent. Inconsistent convergence in chemical compositions during decomposition was observed among different litter species across a range of site conditions, whereas one litter species converged under different climate conditions. Our data clearly demonstrate that plant species rather than climate greatly alters the temporal pattern of litter chemical composition, suggesting the decomposition-chemistry feedback varies among different plant species.

Litter decomposition plays an important role in the terrestrial carbon (C) cycle^1^ and hence the factors controlling its rate are important to understand. Much of the previous work on this process has examined the effect of initial substrate quality^2^,^3^. However, during the course of decomposition, which takes years to decades, there are potential feedbacks between decomposition and litter chemical composition with decomposition altering chemical composition that in turn alters the decomposition rate. These feedbacks lead to a general slowing of the decomposition rate^4^ and the enrichment of certain chemical constituents such as lignin and waxes which may lead to an eventual convergence of chemical composition^3^,^5^,^6^,^7^,^8^,^9^.

The controls of short- and long-term litter decomposition have been extensively studied at various spatial scales^1^,^10^,^11^,^12^,^13^,^14^. At the global scale, the primary controls of litter decomposition are climate variables such as temperature and moisture, the secondary controls include initial litter quality (physical properties, nutrient contents and organic composition), and the tertiary controls are site chemistry and soil biota^1^,^2^,^3^,^12^,^15^,^16^. At finer spatial scales within a particular climatic region where climate is less variable, initial litter chemistry becomes the primary control of litter decomposition, with site properties such as soil type, biochemical status and soil biota potentially becoming more important^13^,^17^,^18^.

Given the potential feedback between decomposition and litter chemistry, studies on the changes in chemical composition during litter decomposition are needed to fully understand the decomposition process and associated controlling mechanisms. Litter chemistry is generally regarded to be closely associated with the structure, turnover and stability of soil organic mater (SOM)^17^,^19^, decomposer community structure and microbial activity^20^, and soil nutrient cycling^21^. In contrast to effects of initial litter quality on decomposition, there is less work on the long-term decomposition-chemistry feedback.

Inconsistent results regarding the changes in chemical composition during litter decomposition have been previously reported. Some researchers suggested that the chemistry of different litter types converged during decomposition^7^, which has been supported by many results of litter bag studies^3^,^6^,^8^,^9^. Others argued that the difference in initial chemical composition among different litter types would have persistent effects on the litter chemical composition after decomposition^13^,^22^. Moreover, different chemical composition pathways could occur, even within one litter type, due to the effects of different decomposition communities and enzyme activities^13^,^16^. The discrepancy among different studies could be attributed to the variations in litter type, soil type, site environmental factors, duration of litter decomposition, or the analytical method used^13^,^14^,^18^.

As noted above, one of the major challenges in understanding changes in litter chemistry during decomposition is related to methodology. Although proximate analysis has been routinely used to determine the chemical compositions of litters^23^,^24^, the correspondence between these operationally determined forms of C (i.e., resistance to hydrolysis in acid) and the actual forms (i.e., lignin) is often indirect and sometimes misleading. For instance, lignin determined on the basis of resistance to hydrolysis in heated acid solutions may potentially include or actually be other materials, such as cutin and tannin^25^. Recently, some high-resolution analytical techniques, such as solid-state nuclear magnetic resonance (NMR), pyrolysis-gas chromatography/mass spectrometry (py-GCMS) and near-infrared spectrometry, have been adopted to characterize the chemical composition of litter samples^3^,^13^,^14^. Organic chemical structures of litter have been characterized primarily through ^13^C cross polarization/magic angle spinning (CP/MAS)^6^,^26^,^27^, and occasionally by^13^C direct polarization/magic angle spinning (DP/MAS)^3^ NMR techniques. Some indices, such as the alkyl C to O-alkyl C (A/O-A) ratio and carbohydrate C to methoxy C (CC/MC) ratio, have been calculated from the NMR spectra to describe the extent of chemical changes in litter decomposition^6^,^26^,^28^.

Despite the potential of NMR, the broad peaks typically associated with ^13^C CP/MAS or DP/MAS spectra of litter present a challenge in identifying specific types of C involved^3^,^17^. Recently a series of advanced spectral editing techniques have been developed and applied for systematically elucidating the structure of complex organic matter^29^. For example, the^13^C chemical shift anisotropy (CSA) filter technique is capable of selecting sp^3^-hybridized C, hence overlapping signals in the 90–110 ppm region attributed to anomeric groups (representing carbohydrates)^30^. In addition, dipolar dephasing technique can separate methoxyl C (characteristic of lignin) from NCH C (characteristic of proteins and peptides), despite that their signals present in the same spectral region (60–45 ppm). These spectral editing techniques enable one to obtain more details in structural changes associated with litter decomposition, and more accurate estimates of how decomposition alters litter chemical composition.

In the present study, we employed several advanced ^13^C NMR spectral editing techniques to investigate the changes in chemical composition in foliar litters during long-term decomposition. Undecomposed and decomposed litter samples were collected from the Long-term Intersite Decomposition Experiment Team (LIDET) project in which the decomposition of 30 species of litter was investigated at 27 sites by a standardized method^4^. The litter samples of four species decomposed for up to 10 years at four sites with different mean annual temperatures (MAT) and mean annual precipitation (MAP) (from Arctic to tropical forest) were used in the present study. The objectives of this study were to study the effects of plant species and climate on the changes of litter chemical composition during long-term decomposition. Specifically, we tested the following hypotheses: (1) both plant species and climate influence the changes in litter chemical composition during decomposition; (2) litter chemical convergence occurs during decomposition due to the recalcitrance of certain compounds and the creation of others by decomposers; and (3) the cumulative mass loss of decomposed litters is related to the degree of change in litter chemical composition.

## Results

### NMR spectroscopy of undecomposed litter samples

**Figure 1:**
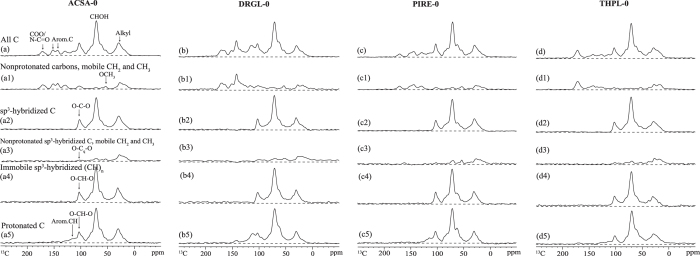
Spectral editing for undecomposed *Acer saccharum* (ACSA), *Drypetes glauca* (DRGL), *Pinus resinosa* (PIRE) and *Thuja plicata* (THPL); (**a–d**) full CP/TOSS spectra for reference with a contact time of 1 ms; **(a1–d1)** dipolar dephasing spectra showing nonprotonated C and mobile segments such as CH_3_ and OCH_3_ with 40 μs dephasing time; **(a2–d2)** selection of alkyl C with a ^13^C CSA filter, which in particular identifies OCO-C typical of sugar rings (CSA filter time = 47 μs); **(a3–d3)** selection of nonprotonated and mobile alkyl C with a CSA filter and dipolar dephasing, which in particular identifies OC (RR’)O-C (CSA filter time = 47 μs; dipolar dephasing time = 40 μs); **(a4–d4)** selection of protonated alkyl C with a CSA filter and short CP, in particular OCHO around 100 ppm (CSA filter time = 47 μs; CP time = 50 μs); **(a5–d5)** selection of protonated-C-only groups.

The NMR peaks were assigned to the major components in undecomposed litters based on spectral-editing spectra and previous NMR studies of litter^29^,^33^,^34^. The CP/TOSS spectra (Fig. 1a–d) of four undecomposed litters were all dominated by signals from oxygenated aliphatic C from carbohydrates and assigned as follows: ~72 ppm, OCH groups (C2, C3 and C5 of pyranoside rings in cellulose and hemicellulose); ~62 ppm, OCH_2_ groups (C6-C); ~82 ppm, OCH groups (C4-C) and ~103 ppm, anomeric (O-C-O) C (C1-C). Anomeric C was separated from the aromatic C at 109 ppm in the ^13^C CSA-filtered spectra (Fig. 1a2–d2). These assignments can also be assisted by the spectra after ^13^C CSA filter and dipolar dephasing (Fig. 1a3–d3)^29^ as well as spectra after ^13^C CSA filter and dipolar dephasing and short CP (Fig. 1a4–d4).

The signal intensity from alkyl C region (46–0 ppm) was the second highest for four undecomposed litter species, with the peak at 29 ppm assigned to CCH_2_C and the signals below 24 ppm due to CH_3_ groups, as well as shoulders at 35 ppm attributed to CH or quaternary C. These signals mainly arose from waxes such as cutin^3^. Note that the shoulder at 22 ppm was due to terminal -CH_3_ of CH_3_COO of hemicelluloses. The signals in the region (60–46 ppm) included the contributions from both methoxyl C (OCH_3_) from lignin at 56 ppm, and N-alkyl C (NCH) (partially from peptides or proteins). The signals of the former group were selectively retained in the dipolar dephasing spectra (Fig. 1a1–d1).

Lignin/tannins were main contributors to signals in regions of aromatic C-C+/H (i.e. non-oxygenated olefinic/aromatic C, 138–109 ppm), and the oxygenated aromatic C-O (140–160 ppm). The broad peaks in aromatic region were characteristic features of guaiacyl lignin: 115 ppm, aromatic CH (G_5_); and 131 ppm, nonprotonated aromatics G_1(ne)_. Note that the “ne” subscript referred to “non-etherified”. Immobile protonated carbon selection spectra (Fig. 1a5–d5) which exhibit the signals of aromatic CH, OCHO, and CHOH also confirmed the presence of aromatic CH (G_5_) at 115 ppm. Assisted by dipolar dephasing spectra, aromaticC-C+/H could be divided into nonprotonated aromatics (aromatic C-C) and protonated aromatics (aromatic C-H). The predominance of condensed tannins was indicated by a sharp aromatic peak at 131 ppm, and more importantly, by the split peak at 145 and 155 ppm in the aromatic C-O region^3^. For lignin, a dominance of guaiacyl lignin was indicated by a peak at 148 ppm together with a shoulder at 153 ppm; a peak at 153 ppm with higher intensity than 148 ppm would suggest the presence of syringyl lignin^25^.

The peak with a maximum at 172 ppm was attributed to carboxyl/carboxylate/amide C (COO/N-C=O) groups. The presence of NCH and amide C were partly associated with proteins/peptides; carboxyl (COO) groups plus CH_3_ were associated with CH_3_COO in hemicellulose. In addition, some COO groups were partially due to fatty acids.

### Comparison of chemical composition of undecomposed litter samples

The ^13^C DP/MAS and DP/MAS dipolar dephasing (DD) spectra of undecomposed *Drypetes glauca* provided quantitative information of chemical constituents (Supplementary Fig. 1). *Pinus resinosa* had the highest abundances of O-alkyl C (i.e., carbohydrates), while *Drypetes glauca* had the lowest amount; the other two species had slightly lower amounts than *Pinus resinosa* (Supplementary Table 2). The amounts of anomeric C (also associated with carbohydrates) for four species were very similar (Supplementary Table 2). The small content of nonprotonated anomeric C indicated that anomeric C of four species was primarily in a protonated form (O-CH-O). This was also confirmed from Fig. 1a3–d3 in which O-C_q_-O peaks (quaternary anomeric C) were inconspicuous. *Thuja plicata* had the largest amount of alkyl C (waxes), whereas *Drypetes glauca* had the smallest amount, 10% lower than *Thuja plicata*. All four undecomposed litters contained methoxyl C (Fig. 1a1–d1); however, the amounts were very small. *Drypetes glauca* had the highest amount of aromatic C-C+/H (Supplementary Table 2) as well as aromatic CH (G5) (Fig. 1a5–d5). For all four litter species, nonprotonated aromatic C accounted for a larger fraction than protonated ones (Supplementary Table 2). The peaks for *Pinus resinosa* and *Thuja plicata* in the aromatic C-O region were consistent with the presence of guaiacyl lignin, confirming that they contained primarily the G unit of lignin found in gymonosperms. The low intensity of aromatic signal for *Thuja plicata* was consistent with its lowest amount of methoxyl C (Supplementary Table 2), suggesting that the lignin content of this species may be low.

On the other hand, the partially split peaks at ~144 and ~154 ppm for *Acer saccharum* indicated the presence of tannins. These split peaks for *Drypetes glauca* were not as well-resolved as those for *Acer saccharum*, suggesting that the former may contain smaller amounts of tannins. No split peaks were observed for *Pinus resinosa* and *Thuja plicata*. As angiosperms, the higher intensity of peaks at 153 ppm of *Acer saccharum* and *Drypetes glauca* suggested that these two species may contain more syringyl lignin than *Pinus resinosa* and *Thuja plicata*. *Acer saccharum* and *Drypetes glauca* contained higher contents of aromatic C-C and aromatic C-O than *Pinus resinosa* and *Thuja plicata* as well (Supplementary Table 2).

### Changes in chemical constituents of litters with decomposition

**Figure 2:**
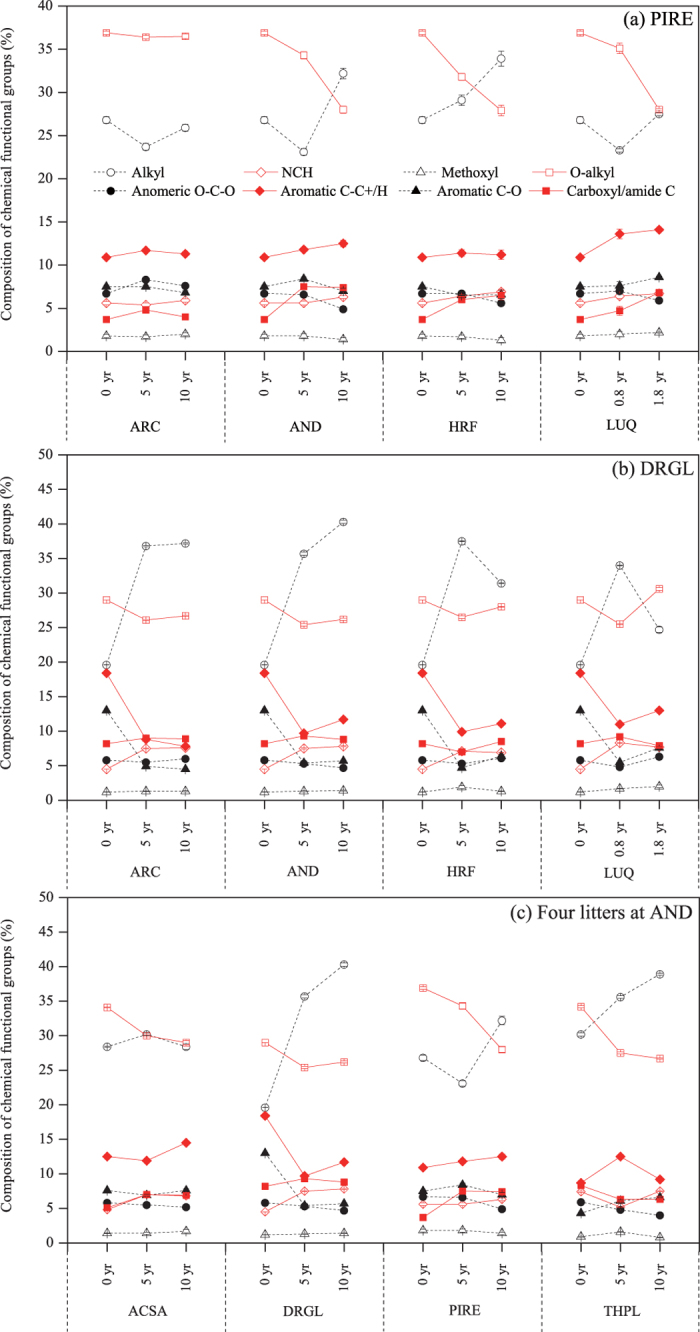
Composition of chemical functional groups (%) in (**a**) *Pinus resinosa* (PIRE) at four sites, (**b**) *Drypetes glauca* (DRGL) at four sites, and (**c**) four litters at H. J. Andrews Forest (AND) obtained by ^13^C CP/TOSS and spectral editing technique. Error bars represent the level of S/N ratio.

The trends in abundances of chemical functional groups in *Pinus resinosa* that were decomposed for 5 and 10 years at ARC, HRF and AND, and for 0.8 and 1.8 years at LUQ were shown in Fig. 2a. Decomposed *Pinus resinosa* at the coldest site, ARC, showed relatively constant chemical compositions after 5- and 10-year decomposition; the extent of changes in all chemical functional groups were ca. 1% except for a decrease in alkyl C of 3.1% after 5-year decomposition. Specifically, *Pinus resinosa* exhibited a decrease in O-alkyl as well as alkyl C but an increase of carboxyl/carboxylate/amide C after 5-year decomposition. After 10 years at AND, *Pinus resinosa* continued to lose O-alkyl C by 8.9% in total, but alkyl C increased by 5.4% relative to its initial abundance in undecomposed litters. Anomeric C started to decrease at this stage, but the change was relatively small. The contents of all other chemical functional groups of this species at this site remained generally constant. At the HRF, *Pinus resinosa* litter followed a similar decomposition pattern to that at AND, but showed larger changes in O-alkyl and alkylC abundances. Despite only two years of decomposition of *Pinus resinosa* at LUQ, there was a similar degree of decrease in O-alkyl C and anomeric C to 10 years of decomposition at AND and HRF. In contrast to the AND and HRF sites there appeared to be almost no increase in alkyl C at LUQ. Another difference was that aromatic C-C+/H was more enriched at LUQ than at the other sites.

**Figure 3:**
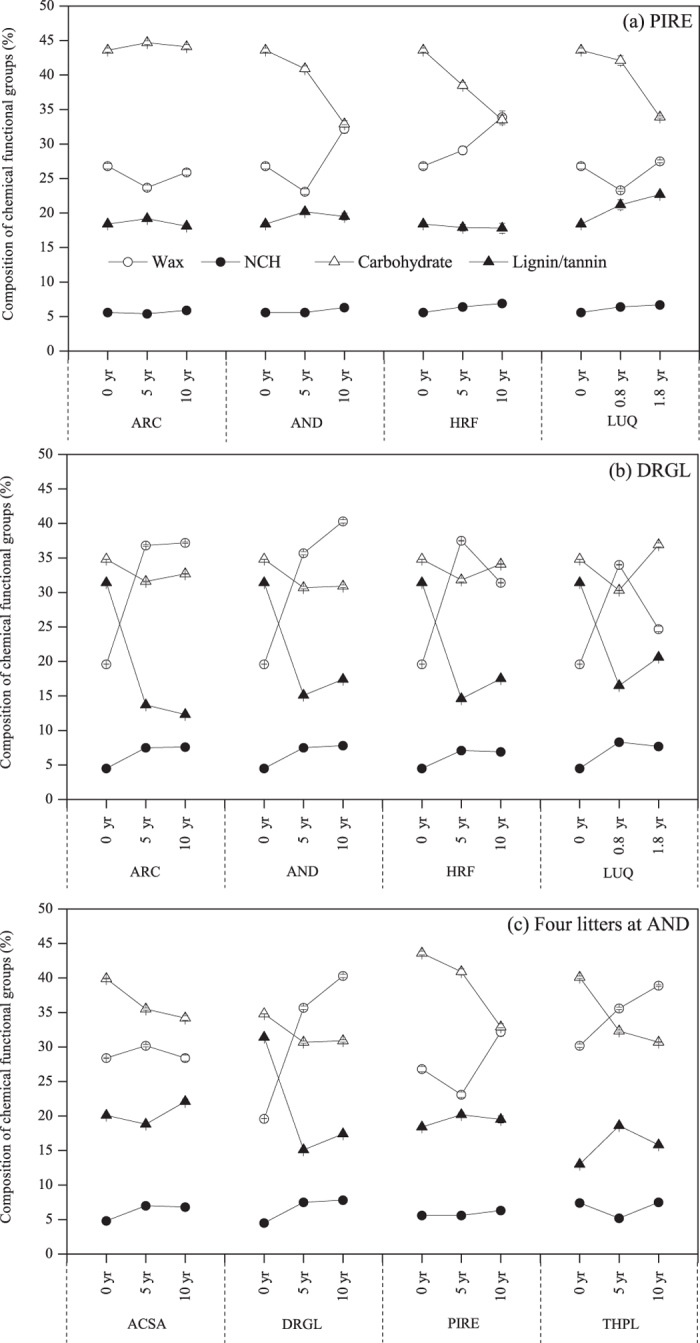
Relative C abundance of different organic compounds (%) in (**a**) *Pinus resinosa* (PIRE) at four sites, (**b**) *Drypetes glauca* (DRGL) at four sites, and (**c**) four litters at H. J. Andrews Forest (AND) obtained by ^13^C CP/TOSS and spectral editing technique. Error bars represent the level of S/N ratio.

The relative C abundance of waxes for *Pinus resinosa* decreased at year 5 and 10 at ARC, at year 5 at AND, and at year 0.8 at LUQ, but increased in all other samples (Fig. 3a). Carbohydrates decreased in all decomposed samples, except for a slight increase for samples at ARC site (Fig. 3a). Lignins/tannins either did not change or increased as decomposition proceeded. Proteins/peptides were slightly enriched (Fig. 3a).

*Drypetes glauca* had a different pattern of compositional change than *Pinus resinosa* (Fig. 3a,b). Compared with the undecomposed litter, decomposed *Drypetes glauca* litters exhibited a progressive enrichment in alkyl C and a decline in relative percentages of aromatic C-C+/H and aromatic C-O, indicating the loss of lignin/tannins. The decrease of O-alkyl C and anomeric C associated with carbohydrates was not as remarkable as that of lignin/tannins. Moreover, the relative intensity of methoxyl groups remained unchanged, whereas NCH C was enriched during decomposition. *Drypetes glauca* became significantly more enriched in waxes and proteins/peptides after decomposition than observed in decomposed *Pinus resinosa* samples (Fig. 3a,b). Contrary to *Pinus resinosa* samples, lignin/tannins clearly decreased in *Drypetes glauca*, with less than half of the initial amount in some samples (Fig. 3b). Carbohydrates generally became less abundant with the exception of a slight increase after 1.8 years at LUQ (Fig. 3b).

The changes in composition of chemical functional groups of four litters at AND had a tendency of an enrichment of alkyl C (except for the *Acer saccharum*) and a decrease of O-alkyl C, while the changes of other functional groups were inconsistent (Fig. 2c). The trends in the change of aromatic C-O were inconsistent for all four species during decomposition, i.e., enrichment for *Thuja plicata*, reduction for *Drypetes glauca*, and generally no change for *Acer saccharum* and *Pinus resinosa* (Fig. 2c). At AND alkyl C were enriched in all decomposed samples except for *Pinus resinosa* at the fifth year and *Acer saccharum* at the tenth year. *Drypetes glauca* had the largest enrichment of alkyl C after decomposition. The relative intensity of NCH groups increased slightly for all species except for *Thuja plicata* at the fifth year. The content of methoxyl C remained unchanged. Both O-alkyl and anomeric C, especially protonated anomeric C, became less abundant during decomposition at AND (Fig. 2c), suggesting that carbohydrates may be decomposed (Fig. 3c).

### Convergence of litter chemistry during decomposition

PCA was applied to evaluate the effects of litter quality on the degradation of four species at AND. The relative importance of each chemical functional group to the first three principal components (PCs) was obtained (Supplementary Table 3). The first component (PC1) accounted for 52.1% of total variance and was dominated by high negative loadings of alkyl (−0.918) and NCH (−0.851) and positive loadings of anomeric C-C+/H (0.781), aromatic C-O (0.760), O-alkyl (0.673) and aromatic C-C+/H (0.666) (Supplementary Table 2). The largest positive and negative loadings for PC2 (23.0% of variance) were aromatic C-C+/H (0.704) and O-alkyl (–0.663), respectively (Supplementary Table 3).

**Figure 4:**
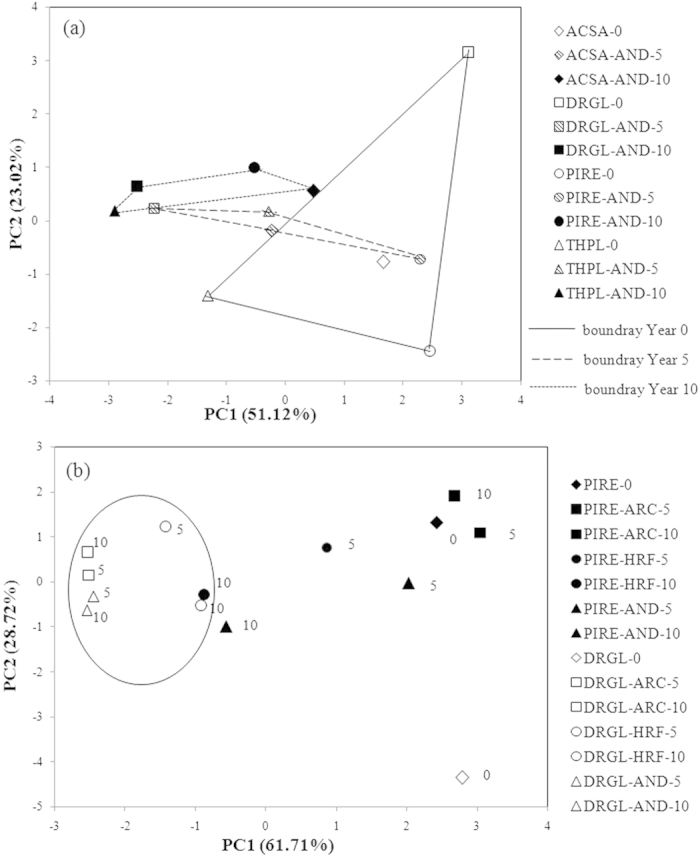
Principle component analysis of the composition of chemical functional groups for (**a**) four species, including *Acer saccharum* (ACSA), *Drypetes glauca* (DRGL), *Pinus resinosa* (PIRE) and *Thuja plicata* (THPL), at H. J. Andrews Forest (AND) at decomposition year of 0, 5 and 10 and (**b**) *Pinus resinosa* (PIRE) and *Drypetes glauca* (DRGL) at three sites, including Arctic Lakes (ARC), H. J. Andrews Forest (AND) and Harvard Forest (HRF), at decomposition year of 0, 5 and 10.

The enclosed areas of 0-year litters (undecomposed litters) were large, indicating diverse initial litter composition (Fig. 4a). As decomposition proceeded, most sample points approached lower PC1 scores, indicating the enrichment of waxes and proteins/peptides (Fig. 4a). The areas enclosed by the four decomposed litters at 5-and 10-year decomposition became smaller than that enclosed by 0-year litters and the shape of enclosed areas also changed, becoming less variable in the PC2 axis. This indicated that aromatic C-C+/H became less abundant for *Drypetes glauca* but enriched in the other three litters. In contrast, the enclosed area remained fairly variable on the PC1 axis (Fig. 4a).

PCA was conducted for two species (*Pinus resinosa* and *Drypetes glauca*) at three sites (ARC, HRF and AND) at decomposition years of 0, 5 and 10 (Fig. 4b). The first component (PC1) accounted for 61.7% of the total variance and was dominated by a negative loading for alkyl (–0.951) and NCH (–0.943), positive loading for O-alkyl (0.895) and smaller loading for anomeric (0.765), aromatic C-O (0.733) and aromatic C-C+/H (0.616) C (Supplementary Table 4). The largest loadings for PC2 (28.7% of variance) were negative loadings of aromatic C-C+/H and aromatic C-O and positive loadings of methoxyl C (Supplementary Table 4).

*Drypetes glauca* and *Pinus resinosa* showed a general convergence in terms of composition. Undecomposed *Pinus resinosa* and *Drypetes glauca* litters had relatively high PC1 scores (Fig. 4b) because of their high content of O-alkyl C and low content of alkyl and NCH C. Undecomposed *Drypetes glauca* was separated from undecomposed *Pinus resinosa* by the low PC2 scores of *Drypetes glauca*, due to the high aromatic C-C+/H and aromatic C-O of *Drypetes glauca*. With increasing decomposition time, the sample points of *Drypetes glauca* and *Pinus resinosa* approached lower PC1 score, except for *Pinus resinosa* at ARC. In addition, except for *Pinus resinosa* at ARC, *Drypetes glauca* moved to higher PC2 score while *Pinus resinosa* moved to lower PC2 score. This result might be due to the significant decrease of aromatic C-C+/H and aromatic C-O for *Drypetes glauca* but inconsistent changes of aromatic C-C+/H and aromatic C-O for *Pinus resinosa*. Decomposed *Drypetes glauca* at AND and ARC at 5-year and 10-year were very close in PCA space, indicating similar compositions. The points of decomposed *Drypetes glauca* at all sites assembled in the circle indicated that the composition converged over time regardless of the climate. Although *Drypetes glauca* at HRF at 10-year moved back toward undecomposed sample composition, this may not have been a significant shift. On the other hand, the pattern of decomposed *Pinus resinosa* samples in PCA space was not the same as for *Drypetes glauca*. At ARC, *Pinus resinosa* decomposed at 5- and 10-years were close to their undecomposed sample in PCA space. This was not true for *Pinus resinosa* at AND and HRF; these samples moved further away from the undecomposed samples after decomposition at a longer time. At AND and HRF, *Pinus resinosa* at 5-year had relatively large difference on PC1 score, but became closer to each other at 10-year. This indicated the composition of both *Pinus resinosa* and *Drypetes glauca* converged regardless of climates if the results for *Pinus resinosa* at ARC was set aside.

### Relationship between cumulative mass losses and compositional changes

**Figure 5:**
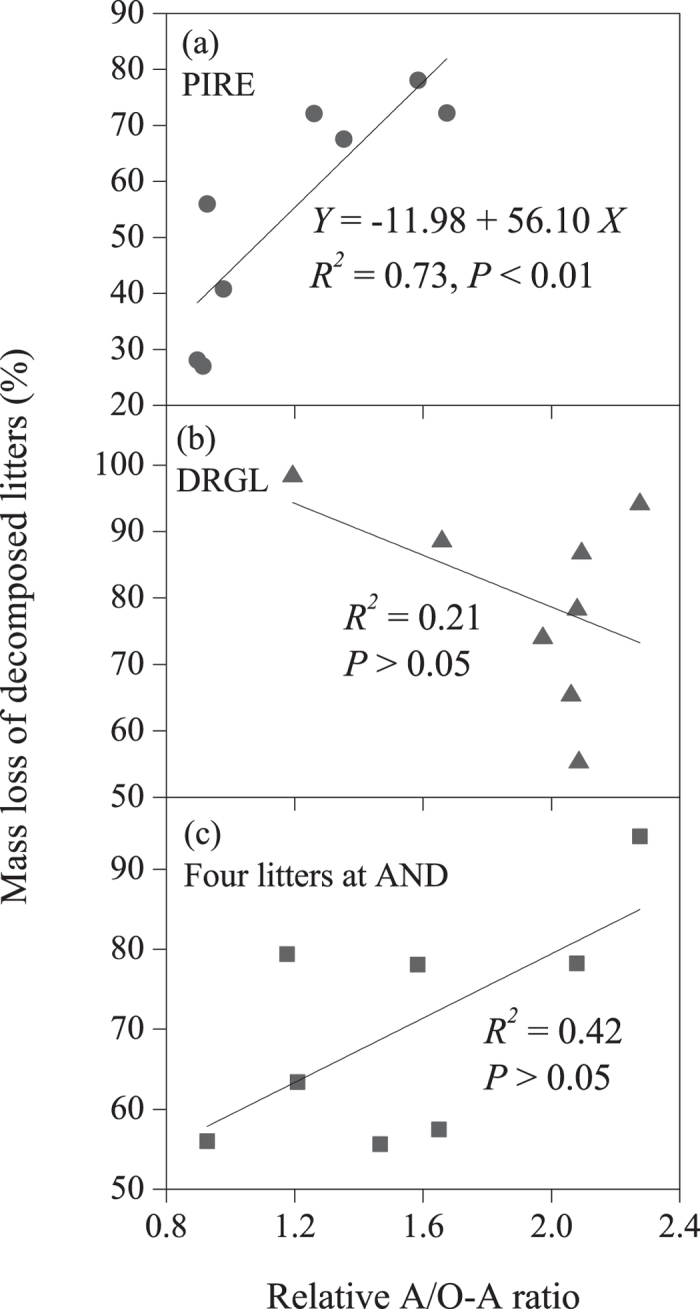
Relationship between the relative alkyl C/O-alkyl C (A/O-A) ratio and mass loss in decomposed litter samples for (**a**) *Pinus resinosa* (PIRE) at four sites, (**b**) *Drypetes glauca* (DRGL) at four sites, and (**c**) four litters at H. J. Andrews Forest (AND).

The alkyl C and O-alkyl C were two chemical functional groups that have greater changes as compared to other chemical functional groups (Fig. 2) suggesting the relative A/O-A ratio could be used as an index reflecting the extent of chemical composition change. A positive correlation (*P* < 0.01) between relative A/O-A and mass loss during decomposition was found in *Pinus resinosa* (Fig. 5a), but not in *Drypetes glauca* (Fig. 5b). Such correlation was not observed in the four litters at AND (Fig. 5c).

## Discussion

### Changes of litter chemical composition during decomposition

Solid-state NMR technique has been extensively used to investigate the changes in litter chemical composition during decomposition in the past two decades^3^,^17^,^27^,^35^. In the present study, we used advanced ^13^C solid-state NMR technique with several spectral editing techniques to obtain the detailed information of chemical composition of four litter species, which were decomposed in 4 different sites. The most significant changes in litter chemical compositions in the present study were an enrichment of alkyl C and a loss of O-alkyl C after decomposition. Previous studies also showed the increased alkyl C content in bamboo leaf litter^36^, maize and wheat straws^8^, black pine needle litter^24^, buffel grass (*Cenchrus ciliaris*) and lucerne (*Medicago sativa*) litters^6^, which would be mainly attributed to selective preservation of recalcitrant organic compounds such as waxes and cutins or an enrichment in cross-linking of the long chain alkyl compounds^3^,^17^,^34^,^36^. Numerous previous NMR studies also demonstrated that the O-alkyl C decreased after decomposition^3^,^6^,^36^, because the O-alkyl C compounds in litters mainly include the carbohydrates, which can be easily utilized by microorganisms^3^. In contrast, Almendros *et al.*^33^ and Lemma *et al.*^34^ reported that decreased alkyl C content and increased O- alkyl C content in some types of litters were observed after decomposition. In addition, we found that alkyl C decreased during the intermediate stage of decomposition (i.e., the 5-year at ARC and AND and, 0.8-year at LUQ) (Fig. 2a). This could be due to relatively quick loss of either some cuticular material or lipids in the early stages of decomposition^3^,^33^.

Unlike previous NMR litter decomposition studies, we were able to distinguish and quantify NCH and methoxyl (region of 60–45 ppm), respectively (Fig. 2, Supplementary Table 2). Our results showed that the content and change of the methoxyl C in litter samples were both very small, whereas NCH was present in comparatively larger amounts and showed larger variations during decomposition (Supplementary Table 2). Previous studies attributed the increase of intensity in the region of 60–45 ppm to preservation of methoxyl C and thus hypothesized that the preservation of lignin occurred during decomposition^6^,^34^. However, we found that NCH, instead of methoxyl C, was enriched during decomposition for three of the four species we examined (Fig. 2). We assumed that the enrichment of NCH could be due to formation of new compounds such as bacterial peptidoglycan and fungal chitin in cell walls during the decomposition^6^,^37^. Hobara *et al.*^38^ also found the yields of total hydrolysable amino acids and total hydrolysable amino sugars increased during litter decomposition, and they suggested that decomposition induced a change in litter chemistry from C-rich plant-derived biopolymers to N-rich microbially-derived biochemicals.

Though it has been assumed that lignin is preserved during decomposition^5^,^39^,^40^, the relative amounts of lignin in the present study did not necessarily increase with decomposition and decreased for *Drypetes glauca* (Fig. 2b). This was consistent with other NMR studies^3^,^26^ and contrasted with what has been reported based on proximate analyses^40^. In addition, the assumption that decomposition proceeded towards enrichment of lignin was not supported by the study on δ^13^C changes of litters during decomposition^3^. These contradictory effects regarding the change of lignin content can depend on differences in litter type, soil type, and site environmental factors, or the analytical method used^3^,^13^.

Therefore, our first hypothesis was supported by the results of this study that both plant species and climate markedly influence the changes in litter chemical composition during decomposition.

### Litter chemical convergence during decomposition

Numerous litter bag studies have demonstrated that different litter types with various initial chemical compositions eventually converge towards a similar chemistry after extensive decomposition^3^,^8^,^9^,^41^. However, recently Wickings *et al.*^13^ revealed that the chemistry of different litter types diverged, rather than converged, by using the py-GC/MS technique, and they attributed the divergent chemical pathways among different litter types to the differences in decomposer communities^13^. Our results suggested that divergent initial litter chemistry did not necessarily completely converge as decomposition proceeded, but could reach several endpoints (Fig. 4). For four litters at AND, although the areas enclosed by four decomposed litters at 5- and 10-year decomposition became smaller, the shape of enclosed areas also changed as the boundary that enclosed composition after 5- and 10-year decomposition became less variable in the PC2 axis and remained fairly variable on PC1 axis. In contrast, Preston *et al.*^3^ who applied the same method found that chemical composition converged over decomposition with the distance on both PC axes decreasing. In addition, we found that the chemical composition of *Pinus resinosa* and *Drypetes glauca* converged regardless of climate conditions (Fig. 4b), with a stronger case of chemistry convergence for *Pinus resinosa* litter. Therefore, our second hypothesis could be supported by the result of one plant species across different climate conditions. However, it was worth mentioning that inconsistent convergence occurred among different plant litter species across a range of site conditions, particularly when LUQ included. This inconsistency may be attributed to the difference in the soil microbial community, soil invertebrate species, and site environmental factors among different sites^13^,^42^. In addition, Bedford *et al.*^42^ suggested the inconsistent convergence in chemical trajectory were related to the bag/mesh design in the litter decomposition experiment. They explained that different bag/mesh sizes would cause different chemical trajectories, through changing the water content of the decomposing leaf, creating stable habitats for invertebrates in compressed layers, and consequently causing the selective decomposition of plant tissues^42^.

In addition, we found the different change patterns in the relative C abundance of different organic compounds (including waxes, carbohydtrates, lignins/tannins, and proteins/peptides) among different plant litter species (Fig. 3). Therefore, we can conclude the plant species rather than climate greatly altered the temporal pattern of litter chemical composition during long-term decomposition.

It is worth noting that the difference in site conditions (such as soil types, climate conditions, management practices applied) may partially attribute to the inconsistent convergence or divergence in the chemical pathways^8^,^13^,^14^, and thus, temporal patterns of litter chemical composition over long-period decomposition or across large spatial scales could be quite different in various litter species-site combinations.

### Relationship between chemical compositional change and mass losses in decomposed litters

Our results showed that changes in the abundance of alkyl and O-alkyl C were the most consistent and obvious ones during decomposition for all four species, confirming that the A/O-A ratio could be a robust index of the degree of compositional changes^26^,^32^. To eliminate the effects of variation in A/O-A ratio among the undecomposed litter types, we used the relative A/O-A ratio as an index to indicate the degree of compositional changes in this study. The positive correlation between the relative A/O-A ratio and mass loss during decomposition was found in *Pinus resinosa* (Fig. 5a), but not in *Drypetes glauca* (Fig. 5b), and such correlation was not found in the four litters at AND (Fig. 5c). These results lead to a rejection of our third hypothesis that the more mass lost, the greater changes in chemical compositions that would occur. In combination with the inconsistent convergence of chemical pathways caused by different litter types revealed by the present study and results of Wickings^13^, the effect of chemical composition changes on the litter decay rate might be more complicated than previously expected^3^,^9^,^41^. To efficiently estimate the mass loss during litter decomposition, Adair *et al.*^1^ developed a model with three C pools (a rapidly decomposing labile pool, an intermediate pool, and a recalcitrant pool), which could explain approximately 70% of the variation of data in their study. According to our results, we can assume that the systematic deviations of predictions by such a model would be partially attributed to the various temporal pattern of litter chemical composition during long-term decomposition.

In conclusion, our study has made a detailed investigation on the changes of litter chemical composition during long-term decomposition across broad spatial scales by using advanced solid-state ^13^C NMR techniques. Our results showed strong evidence that the temporal pattern of litter chemical composition depended largely on the litter species rather than climate. In addition, we also found that the relationship between litter mass loss and changes of chemical composition were affected by plant species and climate conditions. These findings indicated that the decomposition-chemistry feedback would greatly vary among different plant species, and thus parameters representing some plant traits should be incorporated into models that predict the litter chemical change, especially over a long-term period. Further research should be conducted to elucidate the contributions of various chemical pathways of different litter types to the selective preservation, humification, and stability of soil organic matter.

## Materials and Methods

### The LIDET study and litter sample selection

The LIDET was initiated in 1990 to investigate the impacts of substrate quality and macroclimate on the decomposition of leaf and fine root litters over a 10 year period^4^,^31^. A standardized methodology for measuring litter decomposition was used in LIDET at 27 different sites with various climate conditions to study the decomposition patterns of 30 species of litter during the first decade of decomposition^4^. The undecomposed and decomposed samples in LIDET study provide an opportunity to study the effects of plant species and climate conditions on the temporal pattern of litter chemical composition during the long-term decomposition. Because the analysis using different NMR techniques requires significant instrument time, we selected four plant species from four sites to study the effects of plant species and climate on the temporal pattern of litter chemical composition during long-term decomposition.

Undecomposed and decomposed litter samples of *Drypetes glauca* (DRGL) and *Pinus resinosa* (PIRE) from the Arctic Lakes (ARC), H. J. Andrews Forest (AND), Harvard Forest (HRF), and Luquillo Tropical Forest (LUQ) sites were collected to examine interactions between site climate and litter of plant species effects. Previous results of the LIDET study indicated that these two species were significantly different in initial substrate quality (Supplementary Table 1) and exhibited the most consistent temporal patterns of decomposition, with PIRE generally following a single negative exponential decline and DRGL generally following a dual exponential decline^4^. The four sites represented a strong environmental gradient, with mean annual temperatures (MAT) ranging from −7 to 23 °C (Table 1). While MAT at the AND and HFR were just a few degrees apart (8.6 versus 7.1 °C, respectively), their seasonal distribution of mean annual precipitation (MAP) was quite different, with AND having a distinct dry summer and HFR having a uniform seasonal distribution.

To investigate the effect of plant species on the temporal pattern of litter chemical composition within one climate condition, we also used litter samples of *Acer saccharum* (ACSA) and *Thuja plicata* (THPL) from AND, in addition to DRGL and PIRE, because these two species had different initial chemistry (Supplementary Table 1) and represented a wider range of taxa. The site of AND represented an intermediate environmental condition. Four litters examined were decomposed for 5 and 10 years in ARC, AND and HRF, but only decomposed for 0.8 and 1.8 years in LUQ because of the high rate of decomposition at this site. Mass remaining of four litter species after a period of decomposition was determined. For example, after 5 years’ decomposition, the mass remaining (%) for *Pinus resinosa* in ARC, AND and HRF were 71.9%, 44%, and 27.9%, respectively, and these values were 59.2%, 21.9%, and 27.7%, respectively, after 10 years’ decomposition. In LUQ, after 0.8 and 1.8 years’ decomposition, the mass remaining (%) for *Pinus resinosa* were 73% and 32.4%, respectively (Supplementary Table 5).

### 13C NMR spectroscopy

**S**olid state ^13^C NMR analyses were performed using a Bruker Avance III 300 spectrometer at 75 MHz for ^13^C (300 MHz for ^1^H frequency). Samples were run in a double-resonance probe head using 4-mm rotors. NMR experiments included quantitative ^13^C DP/MAS and DP/MAS plus recoupled dipolar dephasing (DP/MAS/DD), ^13^C cross polarization and total suppression of sidebands (CP/TOSS) and ^13^C CP/TOSS plus dipolar dephasing (DD), ^13^C chemical-shift-anisotropy (CSA) filter, spectral editing of immobile CH_2_ + CH.

The quantitative ^13^C DP/MAS NMR experiments were conducted at a spinning speed of 13 kHz with a 200 s recycle delay and 1024 scans. The 90° ^13^C pulse-length was 4 μs. The recycle delay was determined by the cross polarization/spin-lattice relaxation time/total sideband suppression (CP/T_1_-TOSS) technique to ensure that all C nuclei were more than 95% relaxed^29^. Nonprotonated C and mobile C fractions such as methoxyl (OCH_3_) C and CCH_3_ were quantified after DP/MAS technique with a recoupled dipolar-dephasing delay of 68 μs^29^. The recycle delay and number of scans used were the same as for DP/MAS experiment. Considering the long measuring time and similar types of components for four litter species, DP/MAS and DP/MAS/DD spectra were only collected for undecomposed DRGL. The quantitative data of other samples were obtained by calculation of CP efficiency of specific chemical shift regions.

The ^13^C CP/TOSS/DD was conducted using a ^13^C CP/MAS NMR technique with a spinning speed of 5 kHz, a contact time of 1 ms and a ^1^H 90° pulse-length of ca. 4 μs. Four-pulse total suppression of sidebands (TOSS) was employed before detection. Two-pulse phase-modulated (TPPM) decoupling was applied for optimum resolution during detection. Sub-spectra for nonprotonated and mobile C groups were obtained by combining the ^13^C CP/TOSS sequence with a 40-μs DD. The number of scans of ^13^C CP/TOSS and ^13^C CP/TOSS/DD spectra was 6144 for all litter samples. The recycle delays for ^13^C CP/TOSS and ^13^C CP/TOSS/DD was 1 s.

The ^13^C CSA filter inserted into ^13^C CP/TOSS to select the signals of sp^3^-hybridized C. Specifically, this technique separated signals of anomeric (O-C-O) C in carbohydrate rings from those of aromatic C-C+/H by selectively suppressing the latter with a five-pulse ^13^C CSA filter using a CSA-filter time of 47 μs^30^. Non-protonated anomeric C were obtained by combining this filter with a dipolar dephasing time of 40 μs. The CSA filter was also combined with short CP to give selective spectra of protonated anomeric C. A total of 6144 scans were averaged for spectra of CSA filter, CSA filter coupled with short CP, and CSA filter coupled with DD, with a recycle delay of 1 s.

The spectra for immobile CH_2_ + CH groups were obtained from the difference of two spectra. The first one was a ^13^C CP/TOSS spectrum with a short CP time of 50 μs to emphasize protonated C in immobile segments, and the second ^13^C CP/TOSS spectrum was recorded using a short CP of 50 μs coupled with a 40 μs DD, which only contained the residual signals of quaternary C or mobile segments. The second spectrum was subtracted from the first one, and the difference spectrum represented immobile CH_2_ and CH C, with a small CH_3_ contribution^29^. The number of scans was 6144 for all analyzed samples.

Following our previous publications and related literature^3^,^17^,^29^, the NMR spectra obtained in this study were divided into eight regions representing different chemical environments of a ^13^C nucleus: alkyl C (46–0 ppm), methoxyl C/NCH (60–46 ppm), O-alkyl C (92–60 ppm), anomatic C-C/anomatic C-H (109–92 ppm), aromatic C-C/aromatic C-H (138–109 ppm), aromatic C-O (162–138 ppm), carboxyl/amide (190–162 ppm) and aldehyde/ketone (220–190 ppm). The area in each region was calculated by integration, and the relative contents of different C fractions were obtained. On the basis that one (or more) particular chemical functional group is often characteristic of a specific compound, some chemical functional groups identified by NMR analysis can represent major classes of organic compounds. Alkyl C, O-alkyl C and anomatic C, aromatic C-C+/H and aromatic C-O, NCH were characteristic functional groups of waxes, carbohydrates, lignins/tannins, and proteins/peptides, respectively. Thus, the relative C abundance of waxes, carbohydrates, lignin/tannins, and proteins/peptides were calculated by the corresponding C function groups. The alkyl C/O-alkyl C (A/O-A) ratio has been extensively used to represent the extents of changes in chemical composition^6^,^26^,^32^. To eliminate the effects of variation in A/O-A in the undecomposed litters, the relative A/O-A was used in this study. The relative A/O-A ratio was defined as:

Relative A/O-A ratio = (A/O-A value of the sample after a period of degradation) / (A/O-A value of the undecomposed sample).

### Statistical analysis

All of the statistical analyses were performed using the SPSS software (SPSS 13.0 for windows, SPSS Inc., Chicago, USA). Principal component analysis (PCA) was performed on the composition of chemical functional groups in litters. Linear regression analyze was conducted to determine the relationship between the relative A/O-A ratio and mass loss in decomposed litter samples.

## Additional Information

**How to cite this article**: Li, Y. *et al.* Plant species rather than climate greatly alters the temporal pattern of litter chemical composition during long-term decomposition. *Sci. Rep.* **5**, 15783; doi: 10.1038/srep15783 (2015).

## Supplementary Material

Supplementary Information

## Figures and Tables

**Table 1:** Climatic characteristics and biome types of 4 long-term intersite decomposition experiment team (LIDET) sites.

Site	Code	Latitude	Longitude	Elevation(m)	MAP(cm)	MAT(°C)	AET(cm)	PET(cm)	Biome Type
Arctic Lakes, Alaska	ARC	63°38’N	149°34’W	760	32.7	–7	28.4	42.3	Arctic Tundra
Harvard Forest, Massachusetts	HRF	42°40’N	72°15’W	335	115.2	7.1	85.1	104.1	Temperate Deciduous Forest
H. J Andrews Experimental Forest, Oregon	AND	44°14’N	122°11’W	500	230.9	8.6	76.4	98.2	Temperate Conifer Forest
Luquillo Experimental Forest, Puerto Rico	LUQ	18°19’N	65°49’W	350	336.3	23	123.4	125.9	Humid Tropical Forest
