# Supplementary material for: Plant Species Rather Than Climate Greatly Alters the Temporal Pattern of Litter Chemical Composition During Long-Term Decomposition
Source: Sci Rep. 2015 Oct 30;5:15783. doi: 10.1038/srep15783 (PMC4626799; doi:10.1038/srep15783)
Supplement: Supplementary Information [file srep15783-s1.doc]

**Supplementary Information:**

**Plant species rather than climate greatly alters the temporal pattern of litter chemical composition during long-term decomposition**

Yongfu Li^1, 2, †^, *, Na Chen^2, †^, Mark E. Harmon^3^, Yuan Li^2^, Xiaoyan Cao^2^, Mark A. Chappell^4^, Jingdong Mao^2^, *

^1^ Zhejiang Provincial Key Laboratory of Carbon Cycling in Forest Ecosystems and Carbon Sequestration, Zhejiang A & F University, Lin’an 311300, China

^2^ Department of Chemistry and Biochemistry, Old Dominion University, 4541 Hampton Blvd, Norfolk, VA 23529, USA

^3^ Department of Forest Ecosystems and Society, Oregon State University, Corvallis, OR 97331, USA

^4^ Environmental Laboratory, U.S. Army Corps of Engineers, 3909 Halls Ferry Rd., Vicksburg, MS 39180, USA

^†^ These two authors contributed equally to this paper.

* Corresponding authors:

E-mail: [yongfuli@zafu.edu.cn](mailto:yongfuli@zafu.edu.cn) (Y. Li); Tel.: +86-571-63740889; Fax.: +86-571-637-40889

E-mail: [jmao@odu.edu](mailto:jmao@odu.edu) (J. Mao); Tel.: +1-757-683-6874; Fax.: +1-757-683-4628

Submitted to: *Scientific Reports*

Number of text pages (including this cover page): 6

Number of figures: 1

Number of tables: 5

**Supplementary Table 1** Undecomposed litter, including *Acer saccharum* (ACSA), *Drypetes glauca* (DRGL), *Pinus resinosa* (PIRE) and *Thuja plicata* (THPL), substrate quality indices from Long-term Intersite Decomposition Experiment Team (LIDET) study

| Species | C (%) | N (% af) * | C/N | AUF (% af) ^†^ | AUF/N |
| --- | --- | --- | --- | --- | --- |
| ACSA | 49.8 | 0.81 | 61.5 | 16 | 19.8 |
| DRGL | 47.8 | 1.97 | 24.3 | 11 | 5.6 |
| PIRE | 53.4 | 0.59 | 90.5 | 19.3 | 32.8 |
| THPL | 51.1 | 0.62 | 82.4 | 21.5 | 34.5 |

^*^ af, ash-free

^†^AUF, acid unhydrolysable fraction

**Supplementary Table 2 Quantitative composition of chemical functional groups (%) in four undecomposed litters,** **including *Acer saccharum* (ACSA), *Drypetes glauca* (DRGL), *Pinus resinosa* (PIRE) and *Thuja plicata* (THPL)**

| Litter species | ppm | | | | | | | | | | | | |
| --- | --- | --- | --- | --- | --- | --- | --- | --- | --- | --- | --- | --- | --- |
|  | 220-190 | 190-162 | 162-138 | 138-109 | | | 109-92 | | | 92-60 | 60-46 | | 46-0 |
|  | Aldehyde/  Ketone | Carboxyl/Amide | Arom.  C-O | Arom.  C-C+/H | Arom.  C-C | Arom.  C-H | Anom. | Anom.  C-C | Anom.  C-H | O-alkyl | methoxyl | NCH | Alkyl |
| ACSA | 0.3±0.1* | 5.1±0.2 | 7.6±0.2 | 12.5±0.3 | 10.1±0.2 | 2.4±0.2 | 5.8±0.2 | 0.6±0.1 | 5.2±0.2 | 34.1±0.3 | 1.4 ±0.1 | 4.8 ±0.1 | 28.4±0.2 |
| DRGL | 0.4±0.2 | 8.2±0.3 | 13.0±0.3 | 18.4±0.3 | 14.4± 0.3 | 4.0± 0.1 | 5.8±0.2 | 1.5±0.2 | 4.3±0.1 | 29.0±0.2 | 1.2 ±0.1 | 4.5 ±0.2 | 19.6±0.2 |
| PIRE | 0.1±0.1 | 3.7±0.2 | 7.5 ±0.4 | 10.9±0.3 | 8.2± 0.2 | 2.7±0.2 | 6.7±0.1 | 0.8±0.1 | 5.9±0.1 | 36.9±0.4 | 1.8 ±0.2 | 5.6 ±0.1 | 26.8±0.3 |
| THPL | 0.0±0.1 | 8.3±0.4 | 4.3±0.2 | 8.7±0.3 | 7.2±0.3 | 1.5±0.1 | 5.9±0.2 | 0.6±0.1 | 5.3±0.2 | 34.2±0.5 | 0.9 ±0.1 | 7.4 ±0.2 | 30.2±0.3 |

Arom. C-C+/H: total aromatic C; Arom. C-C: nonprotonated aromatic C; Arom. C-H: protonated aromatic C; Arom. C-C+/H = Arom. C-C + Arom.C-H; Anom. C-C+/H: total anomeric C; Anom. C-C: nonprotonatedanomeric C; Anom.C-H: protonated anomeric C; Anom. C-C+/H = Anom. C-C + Anom. C-H

*The values following “±” represent the level of S/N ratio

**Supplementary Table 3** Principal component analysis based on four species, including *Acer saccharum* (ACSA), *Drypetes glauca* (DRGL), *Pinus resinosa* (PIRE) and *Thuja plicata* (THPL), at site H. J. Andrews Forest (AND) at decomposition year of 0, 5 and 10

| PC | 1 | 2 | 3 |
| --- | --- | --- | --- |
| Eigenvalue | 4.2 | 1.8 | 0.9 |
| % of total variance | 52.1 | 23.0 | 12.2 |
| Cumulative % of variance | 52.1 | 75.1 | 87.3 |
| Eigenvectors |  |  |  |
| Alkyl | -0.918 | -0.111 | -0.357 |
| NCH | -0.851 | -0.167 | 0.293 |
| Methoxyl | 0.590 | -0.247 | -0.337 |
| O-alkyl | 0.673 | -0.663 | 0.222 |
| Anomeric | 0.781 | -0.428 | 0.415 |
| Aromatic C-C+/H | 0.666 | 0.704 | -0.134 |
| Aromatic C-O | 0.760 | 0.574 | -8.269E-05 |
| Carboxyl/carboxylate/amide | -0.413 | -0.540 | 0.638 |

**Supplementary Table 4** Principal component analysis based on *Pinus resinosa* (PIRE) and *Drypetes glauca* (DRGL) at three sites, including Arctic Lakes (ARC), H. J. Andrews Forest (AND) and Harvard Forest (HRF), at decomposition year of 0, 5 and 10

| PC | 1 | 2 | 3 |
| --- | --- | --- | --- |
| Eigenvalue | 4.9 | 2.3 | 0.4 |
| % of total variance | 61.7 | 28.7 | 5.4 |
| Cumulative % of variance | 61.7 | 90.4 | 95.9 |
| Eigenvectors |  |  |  |
| Alkyl | -0.951 | 0.248 | -0.142 |
| NCH | -0.943 | 0.306 | 0.067 |
| Methoxyl | 0.559 | 0.691 | -0.334 |
| O-alkyl | 0.895 | 0.403 | 0.098 |
| Anomeric | 0.765 | 0.447 | 0.436 |
| Aromatic C-C+/H | 0.616 | -0.749 | -0.190 |
| Aromatic C-O | 0.733 | -0.673 | -0.007 |
| Carboxyl/carboxylate/amide | -0.727 | -0.537 | 0.253 |

**Supplementary Table 5** The mass remaining (%) in decomposed litters, including *Acer saccharum* (ACSA), *Drypetes glauca* (DRGL), *Pinus resinosa* (PIRE) and *Thuja plicata* (THPL), at different sits

| Species | Site code | | | | | | | | | | |
| --- | --- | --- | --- | --- | --- | --- | --- | --- | --- | --- | --- |
|  | ARC | |  | AND | |  | HRF | |  | LUQ | |
|  | 5 | 10 |  | 5 | 10 |  | 5 | 10 |  | 0.8 | 1.8 |
| ACSA | – | – |  | 36.58 | 20.59 |  | – | – |  | – | – |
| DRGL | 44.77 | 36.47 |  | 21.76 | 5.89 |  | 13.28 | 11.50 |  | 26.03 | 1.65 |
| PIRE | 71.91 | 59.17 |  | 44.01 | 21.92 |  | 27.87 | 27.73 |  | 72.99 | 32.40 |
| THPL | – | – |  | 44.38 | 42.51 |  | – | – |  | – | – |


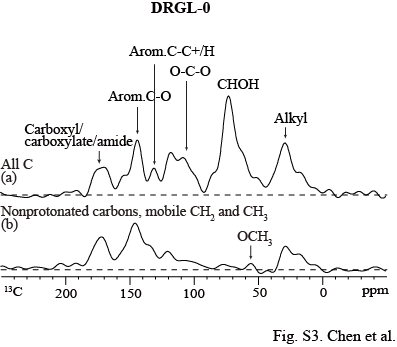


**Supplementary Figure 1** (a) ^13^C DP/MAS and (b) DP/MAS dipolar dephasing spectra of undecomposed *Drypetes glauca* (DRGL) litter
